# The relationship between serum potassium, potassium variability and in-hospital mortality in critically ill patients and a before-after analysis on the impact of computer-assisted potassium control

**DOI:** 10.1186/s13054-014-0720-9

**Published:** 2015-01-06

**Authors:** Lara Hessels, Miriam Hoekstra, Lisa J Mijzen, Mathijs Vogelzang, Wim Dieperink, Annemieke Oude Lansink, Maarten W Nijsten

**Affiliations:** Department of Critical Care, University of Groningen, University Medical Center Groningen, Hanzeplein 1, Groningen, 9700 RB the Netherlands; Department of Anesthesiology, University of Groningen, University Medical Center Groningen, Hanzeplein 1, Groningen, 9700 RB the Netherlands; Department of Critical Care, Laurentius Hospital, Mgr. Driessenstraat 6, Roermond, 6043 CV the Netherlands

## Abstract

**Introduction:**

The relationship between potassium regulation and outcome is not known. Our first aim in the present study was to determine the relationship between potassium level and variability in (ICU) stay and outcome. The second aim was to evaluate the impact of a computer-assisted potassium regulation protocol.

**Methods:**

We performed a retrospective before-after study including all patients >15 years of age admitted for more than 24 hours to the ICU of our university teaching hospital between 2002 and 2011. Potassium control was fully integrated with computerized glucose control (glucose and potassium regulation program for intensive care patients (GRIP-II)). The potassium metrics that we determined included mean potassium, potassium variability (defined as the standard deviation of all potassium levels) and percentage of ICU time below and above the reference range (3.5 through 5.0 mmol/L). These metrics were determined for the first ICU day (early phase) and the subsequent ICU days (late phase; that is, day 2 to day 7). We also compared potassium metrics and in-hospital mortality before and after GRIP-II was implemented in 2006.

**Results:**

Of all 22,347 ICU admissions, 10,451 (47%) patients were included. A total of 206,987 potassium measurements were performed in these patients. Glucose was regulated by GRIP-II in 4,664 (45%) patients. The overall in-hospital mortality was 22%. There was a U-shaped relationship between the potassium level and in-hospital mortality (*P* <0.001). Moreover, potassium variability was independently associated with outcome. After implementation of GRIP-II, in the late phase the time below 3.5 mmol/L decreased from 9.2% to 3.9% and the time above 5.0 mmol/L decreased from 6.1% to 5.2%, and potassium variability decreased from 0.31 to 0.26 mmol/L (all *P* <0.001). The overall decrease in in-hospital mortality from 23.3% before introduction of GRIP-II to 19.9% afterward (*P* <0.001) was not related to a specific potassium subgroup.

**Conclusions:**

Hypokalemia, hyperkalemia and potassium variability were independently associated with increased mortality. Computerized potassium control clearly resulted in improved potassium metrics.

## Introduction

Potassium homeostasis is frequently disturbed in critically ill patients [1]. Underlying diseases or treatments in intensive care unit (ICU) patients often affect the Na^+^/K^+^-ATPase pump. This pump maintains the potassium gradient and can be influenced by many factors, such as insulin, catecholamine and acid–base status. The long-term potassium balance is regulated mainly by the kidney. Thus, dyskalemia is often the result of renal impairment [1,2].

Both hypo- and hyperkalemia are known to induce potentially lethal arrhythmias and cardiac dysfunction, as well as other complications [1,3,4]. Derangements in serum potassium levels in ICU patients should therefore be avoided, and monitoring of potassium is mandatory.

There are surprisingly few data on the relationship between serum potassium and mortality in ICU patients. A recent study showed a strong, independent association between hyperkalemia at the onset of ICU treatment and in-hospital mortality, even at moderate increases above the normal range. A causal relation could not be demonstrated [5].

Our first objective in the present study was to evaluate the relationship between potassium levels and in-hospital mortality. In 2006, our ICU introduced a nurse-centered, computerized potassium regulation protocol integrated with previously implemented computerized glucose control. Our secondary objective was to evaluate the impact of this computerized protocol on potassium control.

## Materials and methods

### Study population

This retrospective observational cohort study was performed at the adult ICU of our university teaching hospital. This ICU includes three surgical subunits (including cardiothoracic surgery and neurosurgery) and a medical subunit, comprising a total of 47 beds. All patients, ages >15 years who were admitted to the ICU during a 10-year period (2002 through 2011) were evaluated. In order to assess the role of ICU-acquired potassium derangements, only patients admitted for at least 24 hours were studied. If a patient had multiple ICU admissions, the first ICU admission of the patient’s last hospital admission was used for analysis.

The anonymized data analysis in this study was performed in accordance with the guidelines and outlined in Dutch legislation, and the study was approved by the medical ethics committee of our institution (Medisch Ethische Commissie, UMC Groningen, METc 2014.264). Because this was a retrospective study of routinely collected data, informed consent was not required by our ethics committee.

### Potassium measurements and other parameters

Potassium measurements determined before ICU admission, as well as samples known to be hemolyzed or otherwise obviously erroneous and thus considered less reliable, were excluded. For this purpose, the authenticity of all potassium measurements ≥7.0 mmol/L and ≤2.0 mmol/L was also separately verified by examination of patient files. The selected measurements were verified by scanning the patients’ medical records for known causes of extreme serum potassium levels, such as previously diagnosed hypo- or hyperkalemia, renal dysfunction and cardiopulmonary resuscitation during the corresponding hospital admission. When no plausible explanation was found for an extreme measurement and the measurement represented an isolated high or low value, preceded and followed by normal values from samples taken within 2 hours of the abnormal measurement, this measurement was excluded from further analysis.

Data were obtained from our electronic database and patient files and included basic demographics, reason for ICU admission, in-hospital mortality, inclusion in the glucose and potassium regulation program for intensive care patients (GRIP-II) and hospital follow-up. All potassium levels (reference range, 3.5 through 5.0 mmol/L) measured during the patient’s ICU stay, with a maximum of the first 7 days of ICU admission, were collected. A recent recommendation on glucose metrics was used as a guide to decide which potassium values to report [6]. Minimum, maximum and mean potassium levels, as well as potassium variability, were determined for every patient. The minimum and maximum potassium levels of the patients were used to derive the incidence of hypo- and hyperkalemia.

In cases where a patient was both hypokalemic and hyperkalemic, both values were counted. The potassium range was defined as the difference between the minimal and maximal potassium levels. Potassium variability was defined as the standard deviation (SD) of the potassium measurements in every patient. The admission serum potassium level was defined as the first measurement within 24 hours after ICU admission. Mild hypokalemia was defined as <3.5 mmol/L to 3.0 mmol/L, and severe hypokalemia was defined as <3.0 mmol/L. Mild hyperkalemia was defined as >5.0 mmol/L to 6.0 mmol/L, and severe hyperkalemia was defined as >6.0 mmol/L [7]. Potassium levels were measured and recorded in millimoles per liter (1 mmol/L = 1 mEq/L).

Disturbances in renal function were defined and staged according to the Kidney Disease: Improving Global Outcomes (KDIGO) definition of acute kidney injury (AKI) [8]. Severity of illness was defined according to the Acute Physiology and Chronic Health Evaluation II (APACHE II) score when available. Admission serum glucose was defined as the first glucose measurement within the first 24 hours after ICU admission. In order to assess the relation of marked admission hyperglycemia with potassium, hyperglycemia was categorized into 15 to 20 mmol/L and >20 mmol/L groups.

### Computerized potassium regulation protocol

A nurse-centered, computerized potassium regulation protocol called glucose and potassium regulation in intensive care patients (GRIP) has been fully operational at our ICU for several years. This protocol was first implemented as a glucose regulation system (GRIP-I), but a potassium algorithm was successfully integrated later (GRIP-II). GRIP-II provides advice about the desired rate of potassium administration and the time interval until the next potassium measurement after analysis of a blood sample. All recommendations made by GRIP-II can be overruled or adjusted by a nurse or physician at any time, and all were automatically recorded. The potassium target range was set in the middle of the normal range (that is, 4.3 mmol/L), similar to the potassium target before implementation of this computerized protocol. More detailed descriptions of the design and implementation of this system have been published previously [9,10].

### Endpoints

The primary endpoint of this study was in-hospital mortality. The secondary endpoint was the effect of GRIP-II on potassium control.

### Statistical analysis

All potassium measurements were split into an early phase (first ICU day) and late phase (ICU days 2 through 7) for both the whole patient cohort and divided according to the regulation of GRIP-II. Baseline demographics and blood potassium levels were compared between survivors and nonsurvivors and before and after GRIP-II using contingency tables and the χ^2^ test. The categorization of patients by regulation of serum potassium levels by GRIP-II was made by conducting an intention-to-treat analysis.

Logistic multivariate regression analysis was performed to assess the independent relationship between the obtained variables and in-hospital mortality. The regression analysis was corrected for sex, age, severity of illness, AKI, mean potassium, mean potassium squared and potassium variability. A two-sided *P*-value of 0.05 was considered significant. Data reduction and statistical analysis were performed with SPSS version 22 software (IBM SPSS, Chicago, IL, USA).

## Results

During the study period, a total of 22,347 patients were admitted to our ICU, and they had a total of 256,410 serum potassium measurements. Of these potassium measurements, 256,200 (99.9%) were assessed as realistic. Eventually, we had 10,451 patients (46.7%) with an aggregate of 206,987 serum potassium measurements who were admitted to our ICU for more than 24 hours. The data gathered during the first 24 hours of the ICU stay were available for 10,327 patients (98.8%). The minimum and maximum serum potassium levels observed were 1.5 mmol/L and 10.8 mmol/L, respectively. The baseline characteristics of the 10,451 patients studied are shown in Table 1. AKI occurred in 3,443 (33.3%) of the patients. A total of 999 (9.6%) patients received renal replacement therapy (RRT).

**Table 1:** **Patient characteristics and blood summary statistics** ^**a**^

		**Total (** ***N*** **= 10,451)**	**Survivors (** ***n*** **= 8,175)**	**Nonsurvivors (** ***n*** **= 2,276)**	***P*** **-value**
**Baseline characteristics**					
Age, yr, mean (SD)		59.4 (16.7)	58.3 (16.9)	63.3 (15.4)	<0.001*
Sex, male, *n* (%)		6,340 (60.7)	5,007 (61.2)	1,333 (58.6)	0.021
Reason for admission					
Medical		2,766 (27.5)	1798 (21.9)	977 (42.9)	<0.001
Surgical		7,670 (73.5)	6,372 (78.1)	1,298 (57.1)	
Included in GRIP-II		4,664 (44.6)	3,735 (45.7)	929 (40.8)	<0.001
LOS ICU, days		4.1 (2.0 to 10.1)	3.8 (2.0 to 9.3)	5.9 (2.9 to 12.8)	<0.001*
LOS hospital, days		17.8 (10.1 to 32.0)	19.8 (12.1 to 34.8)	9.9 (4.2 to 21.4)	<0.001*
APACHE II score^b^		16 (12 to 21)	15 (11 to 19)	21 (17 to 27)	<0.001*
AKI^c^		3,443 (33.3)	2,162 (26.5)	1,281 (56.3)	<0.001
KDIGO stage 1		1,388 (40.3)	1,033 (47.8)	355 (27.8)	
KDIGO stage 2		680 (19.8)	432 (20.0)	248 (19.4)	
KDIGO stage 3		1,375 (40.0)	697 (31.8)	678 (52.9)	
RRT		999 (9.6)	524 (6.4)	475 (20.9)	<0.001
**Potassium summary statistics, early phase** ^**d**^					
Admission K^+^ level, mmol/L		4.1 (3.7 to 4.5)	4.0 (3.7 to 4.4)	4.1 (3.7 to 4.7)	<0.001*
K^+^ measurements, *n*		6.0 (3.0 to 8.0)	6.0 (3.0 to 8.0)	5.0 (3.0 to 8.0)	0.235*
Mean K^+^ level, mmol/L		4.2 (3.9 to 4.5)	4.2 (3.9 to 4.5)	4.2 (3.8 to 4.6)	0.025*
K^+^ variability, mmol/L		0.29 (0.19 to 0.43)	0.28 (0.19 to 0.42)	0.32 (0.21 to 0.50)	<0.001*
K^+^ range, mmol/L		0.70 (0.40 to 1.10)	0.70 (0.40 to 1.10)	0.80 (0.40 to 1.20)	<0.001*
Time in hypokalemia, mean SD^e^		7.4 (21.4)	6.7 (20.7)	9.8 (23.7)	<0.001*
Time in hyperkalemia, mean SD^e^		7.6 (21.5)	6.5 (19.7)	11.4 (26.7)	<0.001*
Hypokalemia, mild		1,877 (18.2%)	1,417 (17.6%)	460 (20.3%)	0.003
Hypokalemia, severe		418 (4.0%)	272 (3.4%)	146 (6.5%)	<0.001
Hyperkalemia, mild		1,677 (16.2%)	1,218 (15.1%)	459 (20.3%)	<0.001
Hyperkalemia, severe		411 (4.0%)	259 (3.2%)	152 (6.7%)	<0.001
**Potassium summary statistics, late phase**					
Mean K^+^ level, mmol/L		4.2 (3.9 to 4.4)	4.1 (3.9 to 4.4)	4.2 (4.0 to 4.6)	<0.001*
K+ measurements, *n*		2.0 (1.0 to 3.9)	1.9 (1.0 to 3.6)	2.2 (1.1 to 4.5)	<0.001*
K^+^ variability, mmol/L		0.28 (0.19 to 0.40)	0.26 (0.17 to 0.37)	0.35 (0.24 to 0.51)	<0.001*
K^+^ range, mmol/L		0.28 (0.03 to 0.50)	0.25 (0.00 to 0.47)	0.36 (0.10 to 0.60)	<0.001*
Time in hypokalemia, mean (SD)^e^		6.4 (17.6)	6.3 (17.8)	6.7 (16.8)	<0.001*
Time in hyperkalemia, mean (SD)^e^		5.7 (17.0)	3.5 (12.8)	13.4 (25.9)	<0.001*
Hypokalemia, mild		2,110 (20.2%)	1,597 (19.5%)	513 (22.5%)	0.002
Hypokalemia, severe		345 (3.3%)	237 (2.9%)	108 (4.8%)	<0.001
Hyperkalemia, mild		1,733 (17.0%)	1,127 (13.8%)	646 (28.4%)	<0.001
Hyperkalemia, severe		375 (3.6%)	140 (1.7%)	235 (10.3%)	<0.001

^a^GRIP-II, Glucose and potassium regulation program for intensive care patients; LOS, Length of stay; RRT, Renal replacement therapy; SD, Standard deviation. Values are expressed as number (%) or median (interquartile range) unless otherwise specified. Statistical analysis was performed by using a χ^2^ test, unless marked by an asterisk, in which case a Mann–Whitney *U* test was used. ^b^Acute Physiology and Chronic Health Evaluation II (APACHE II) scores were available for 5,294 (50.7%) patients. ^c^Acute kidney injury (AKI) severity was defined on the basis of the Acute Kidney Injury Network’s Kidney Disease: Improving Global Outcomes (KDIGO) criteria [8]. There were no data available for 6 (0.06%) patients. ^d^Potassium levels during the first 24 hours were known for 10,327 (98.8%) patients. ^e^Percentage of total intensive care unit (ICU) stay. Nonsurvivors and survivors differed significantly from each other. Nonsurvivors had more potassium derangements and a higher potassium variability.

### Abnormal serum potassium levels and in-hospital mortality

**Figure 1:**
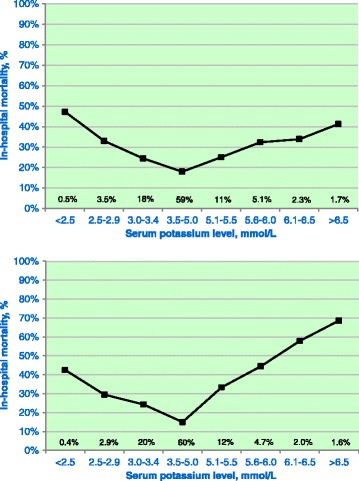
**Lowest and highest potassium levels and outcomes in** **the early and late phases of intensive care unit admission.** Relationship between abnormal potassium levels and mortality during the first 24 hours of intensive care unit (ICU) admission (early phase; upper panel) and days 2 through 7 (late phase; lower panel) of ICU admission. This distinction was made because the initial derangements often cannot be influenced by ICU treatment. Both the lowest and the highest potassium levels measured during the relevant episode were used. Lower and higher potassium levels were both associated with a marked increase in mortality risk. The incidences are indicated above the *x*-axis. Thus, 59% and 60% of the patients had neither hypokalemia nor hyperkalemia in the early and late phases, respectively. Because some patients are represented in both a hypokalemic and a hyperkalemic category, the percentages add up to more than 100%.

**Figure 2:**
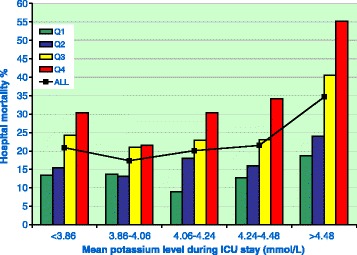
**Relationship of mean potassium level and potassium variability with mortality.** The relationship between mean potassium and mortality is depicted for five quintiles (black curve). For each mean potassium quintile, quartiles of potassium variability (colored bars) are shown.

The in-hospital mortality number was 2,276 (21.8%), and admission potassium levels were higher in patients who died during their hospital stay than among patients who survived. It should be stressed that all the incidences mentioned refer to the number of patients with potassium derangements, not to the number of deranged measurements. There was a U-shaped relationship between potassium levels and in-hospital mortality (*P* <0.001) (Figure 1). Potassium variability was independently related to outcome. The independent impact of variability is given in Figure 2, which shows mean potassium in quintiles and potassium variability in quartiles within each quintile (Table 2). Figure 2 shows evidence of lower in-hospital mortality associated with the lower normal range for potassium, as well as lower mortality associated with lower variability across all quintiles. Overall, we saw a lower potassium variability in survivors in both the early and late phases (*P* <0.001). The design of Figure 2 was copied as faithfully as possible from a figure reported by Krinsley [11] that depicted a very similar phenomenon for mean glucose and glucose variability. Multivariate analysis showed an independent association with in-hospital mortality for the occurrence of both hypokalemia and hyperkalemia and potassium variability with and without inclusion of APACHE II and AKI data (Table 3).

**Table 2:** **Potassium variability quartiles used for each mean potassium quintile shown in Figure** 2

**Mean potassium concentration (mmol/L)**	**Quartile 1**	**Quartile 2**	**Quartile 3**	**Quartile 4**
<3.86 (*n* = 2,089)	<0.17	0.17 to 0.26	0.26 to 0.38	>0.38
3.86 to 4.06 (*n* = 2,089)	<0.18	0.18 to 0.26	0.26 to 0.37	>0.37
4.06 to 4.24 (*n* = 2,111)	<0.19	0.19 to 0.28	0.28 to 0.38	>0.38
4.24 to 4.48 (*n* = 2,080)	<0.19	0.19 to 0.28	0.28 to 0.40	>0.40
>4.48 (*n* = 2,082)	<0.21	0.21 to 0.33	0.33 to 0.51	>0.51
Total = 10,451

**Table 3:** **Multivariate analysis for hospital mortality** ^**a**^

	**OR (95% CI)**	***P*** **-value**
**Model 1**		
Sex, female	1.08 (0.97 to 1.20)	0.159
Age	1.018 (1.014 to 1.021)	<0.001
Mean potassium	0.002 (0.000 to 0.008)	<0.001
Mean potassium squared	2.18 (1.85 to 2.57)	<0.001
Potassium variability	9.37 (7.25 to 12.10)	<0.001
**Model 2**		
Sex, female	1.12 (1.01 to 1.25)	0.032
Age	1.017 (1.013 to 1.020)	<0.001
AKI	2.50 (2.25 to 2.79)	<0.001
Mean potassium	0.003 (0.001 to 0.013)	<0.001
Mean potassium squared	2.02 (1.71 to 2.38)	<0.001
Potassium variability	5.83 (4.49 to 7.58)	<0.001
**Model 3**		
Sex, female	1.22 (1.05 to 1.42)	0.012
Age	1.008 (1.003 to 1.013)	0.002
APACHE II score	1.104 (1.091 to 1.116)	<0.001
AKI	1.76 (1.50 to 2.06)	<0.001
Mean potassium	0.008 (0.001 to 0.082)	<0.001
Mean potassium squared	1.84 (1.40 to 2.41)	<0.001
Potassium variability	5.61 (3.64 to 8.66)	<0.001

^a^CI, Confidence interval; OR, Odds ratio. Data are adjusted for sex, age, acute kidney injury (AKI), severity of illness (Acute Physiology and Chronic Health Evaluation II (APACHE II) score), mean potassium, mean potassium squared and potassium variability observed between 24 hours and 7 days after admission. For all variables except potassium variability (9,228 patients (88%)) and APACHE II score (4,883 patients (51%)), virtually complete data were available, therefore the multivariate analysis was performed with APACHE II score (lower panel) and without APACHE II score. In-hospital mortality was associated with all domains of potassium control. In order to test for a U-shaped relationship of mean potassium with hospital mortality, the mean potassium concentration was both included directly and squared.

Time in hypo- and hyperkalemia was higher for nonsurvivors for both the early and late phases (*P* <0.001) (Table 1). Time in hypo- and hyperkalemia was noted as a mean percentage of the total ICU stay and not as a median percentage, because the medians were 0%. Both mild and severe hypokalemia occurred more often in nonsurvivors than in survivors, during both the early phase and the late phase. The incidence of mild and severe hyperkalemia was also higher in nonsurvivors.

### Abnormal serum potassium levels and in-hospital mortality before and after GRIP-II

**Figure 3:**
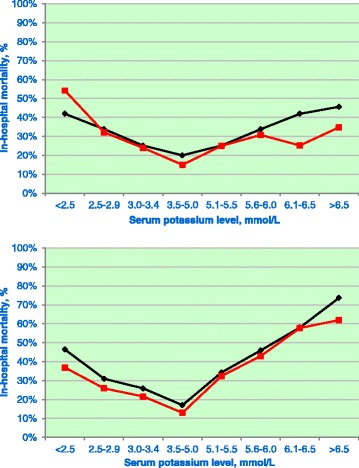
**Relationship between lowest and highest potassium level and outcome during before and after glucose and potassium regulation program for intensive care patients (GRIP-II).** Analogously to Figure 1, here mortality is depicted as a function of abnormal potassium values observed during the early phase (upper panel) and the late phase (lower panel). Patients treated before GRIP-II are shown in black and with GRIP-II in red. Note that, in contrast to the early phase, mortality in the late phase is either comparable or lower in the GRIP-II group across the potassium range.

A total of 4,664 (44.6%) patients were included in GRIP-II. The baseline patient characteristics before and after the introduction of GRIP-II are shown in Table 4. The mean ages (SD) before and after GRIP-II were 59 ± 17 years and 60 ± 16 years, respectively, and 60% and 62% of patients in these two groups, respectively, were male. After implementation of GRIP-II, the number of potassium measurements increased from 1.7 (interquartile range (IQR), 1.1 to 3.0) per patient per day to 5.5 (IQR, 3.5 to 7.3) measurements per patient per day (*P* <0.001). The occurrence of AKI, as well the use of RRT, did not differ before and after the implementation of GRIP-II. More patients with AKI developed mild and severe hyperkalemia (Table 5). Also, patients with marked hyperglycemia at admission more frequently developed hyperkalemia than patients with normoglycemia (Table 6). The overall in-hospital mortality decreased from 1,347 (23.3%) to 929 (19.9%) after implementation of GRIP-II (Table 4). The U-shaped relationship between potassium extremes and mortality persisted after the introduction of GRIP-II (Figure 3).

**Table 4:** **Baseline characteristics and blood potassium summary statistics before and after introduction of GRIP-II** ^**a**^

		**Before GRIP-II**				**After GRIP-II**				
		**Total (** ***N*** **= 5,787)**	**Survivors (** ***n*** **= 4,440)**	**Nonsurvivors (** ***n*** **= 1,347)**	***P*** **-value**	**Total (** ***N*** **= 4,664)**	**Survivors (** ***n*** **= 3,735)**	**Nonsurvivors (** ***n*** **= 929)**	***P*** **-value**	***P*** **-value** ^**b**^
**Baseline characteristics**										
Age, yr, mean (SD)		58.6 (17.1)	57.3 (17.3)	62.6 (15.7)	<0.001*	60.4 (16.2)	59.4 (16.4)	64.2 (14.8)	<0.001*	<0.001
Sex, male		3,453 (59.7)	2,652 (59.7)	801 (59.5)	0.863	2,887 (61.9)	2,355 (63.1)	532 (57.3)	0.001	0.020
Reason for admission										
Medical		1,979 (34.3)	1,270 (28.7)	709 (52.6)	<0.001	787 (16.9)	519 (13.9)	268 (28.9)	<0.001	<0.001
Surgical		3,799 (65.7)	3,161 (71.3)	638 (47.4)	<0.001	3,871 (83.1)	3,211 (86.1)	660 (71.1)	<0.001	<0.001
LOS ICU, days		4.2 (21. to 10.0)	3.9 (2.0 to 9.0)	5.8 (2.9 to 12.3)	<0.001*	4.0 (2.0 to 10.5)	3.8 (1.9 to 9.7)	6.1 (3.0 to 13.4)	<0.001*	0.194*
LOS hospital, days		17.5 (9.9 to 18.3)	19.8 (12.1 to 35.1)	9.3 (4.1 to 20.1)	<0.001*	18.1 (10.3 to 32.5)	19.8 (12.2 to 34.5)	10.3 (4.3 to 23.7)	<0.001*	0.005*
APACHE II score^c^		17 (12 to 22)	15 (11 to 20)	21 (17 to 28)	<0.001*	16 (12 to 21)	15 (11 to 19)	21 (17 to 27)	<0.001*	0.222*
AKI^d^		1,934 (33.4)	1,174 (26.5)	760 (56.4)	<0.001	1,509 (32.3)	988 (26.4)	521 (56.1)	<0.001	0.384
KDIGO stage 1		767 (39.7)	551 (47.0)	216 (28.4)		621 (41.2)	482 (48.8)	139 (26.7)		
KDIGO stage 2		376 (19.4)	229 (19.5)	147 (19.3)		304 (20.1)	203 (20.5)	101 (19.4)		
KDIGO stage 3		791 (40.9)	394 (33.5)	397 (52.2)		584 (38.7)	303 (30.7)	281 (53.9)		
RRT		564 (9.7)	299 (6.7)	265 (19.7)	<0.001	435 (9.3)	225 (6.0)	210 (22.6)	<0.001	0.466
K^+^ measurements/day, *n*		1.7 (1.1 to 3.0)	1.7 (1.1 to 3.1)	1.6 (1.0 to 2.8)	0.003*	5.5 (3.5 to 7.3)	5.4 (3.6 to 7.2)	5.6 (3.0 to 8.1)	0.032*	<0.001*
**Potassium summary statistics, early phase** ^**e**^										
Admission K^+^ level, mmol/L		4.1 (3.7 to 4.5)	4.1 (3.7 to 4.5)	4.2 (3.7 to 4.7)	<0.001*	4.0 (3.7 to 4.4)	4.0 (3.7 to 4.4)	4.1 (3.7 to 4.6)	0.001*	<0.001*
Mean K^+^ level, mmol/L		4.1 (3.8 to 4.5)	4.1 (3.8 to 4.5)	4.2 (3.8 to 4.7)	<0.004*	4.2 (3.9 to 4.5)	4.2 (3.9 to 4.4)	4.2 (3.8 to 4.5)	0.935*	0.007*
K^+^ variability, mmol/L		0.30 (0.17 to 0.47)	0.29 (0.16 to 0.45)	0.33 (0.18 to 0.53)	<0.001*	0.29 (0.20 to 0.40)	0.28 (0.19 to 0.39)	0.32 (0.22 to 0.45)	<0.001*	0.105*
K^+^ range, mmol/L		0.50 (0.20 to 1.00)	0.5 (0.20 to 0.90)	0.6 (0.30 to 1.1)	<0.001*	0.80 (0.60 to 1.20)	0.8 (0.60 to 1.20)	0.9 (0.70 to 1.40)	<0.001*	<0.001*
Time in hypokalemia, mean (SD)^f^		8.9 (25.3)	8.2 (24.6)	11.0 (25.3)	<0.001*	5.5 (15.0)	4.9 (14.4)	8.0 (16.9)	<0.001*	<0.001*
Time in hyperkalemia, mean (SD)^f^		8.1 (23.3)	6.8 (21.3)	12.0 (28.5)	<0.001*	7.0 (19.0)	6.1 (17.6)	10.5 (23.8)	<0.001*	<0.001*
Hypokalemia, mild		964 (16.9%)	772 (16.5%)	242 (18.0%)	0.201	913 (19.8%)	695 (18.8%)	218 (23.7%)	0.001	<0.001
Hypokalemia, severe		226 (4.0%)	147 (3.4%)	79 (5.9%)	<0.001	192 (4.2%)	125 (3.4%)	67 (7.3%)	<0.001	0.590
Hyperkalemia, mild		850 (14.9%)	612 (14.0%)	238 (17.7%)	0.001	827 (17.9%)	606 (16.4%)	221 (24.1%)	<0.001	<0.001
Hyperkalemia, severe		227 (4.0%)	128 (2.9%)	99 (7.4%)	<0.001	184 (4.0%)	131 (3.5%)	53 (5.8%)	0.002	0.961
**Potassium summary statistics, late phase**										
Mean K^+^ level, mmol/L		4.1 (3.8 to 4.4)	4.1 (3.8 to 4.3)	4.2 (3.9 to 4.6)	<0.001*	4.2 (4.0 to 4.4)	4.2 (4.0 to 4.4)	4.3 (4.1 to 4.6)	<0.001*	<0.001*
K^+^ variability, mmol/L		0.31 (0.20 to 0.46)	0.29 (0.19 to 0.42)	0.38 (0.25 to 0.55)	<0.001*	0.26 (0.18 to 0.35)	0.24 (0.17 to 0.32)	0.33 (0.23 to 0.44)	<0.001*	<0.001*
K^+^ range, mmol/L		0.10 (0.00 to 0.33)	0.1 (0.00 to 0.30)	0.18 (0.00 to 0.4)	<0.001*	0.43 (0.30 to 60)	0.40 (0.25 to 0.57)	0.55 (0.4 to 0.75)	<0.001*	<0.001*
Time in hypokalemia, mean (SD)^f^		9.2 (20.9)	9.1 (21.4)	9.3 (19.4)	<0.001*	3.0 (11.3)	3.0 (11.4)	3.0 (10.7)	0.060*	<0.001*
Time in hyperkalemia, mean (SD)^f^		6.1 (18.2)	3.7 (13.6)	13.9 (27.2)	<0.001*	5.2 (15.5)	2.2 (11.8)	12.7 (23.9)	<0.001*	<0.001*
Hypokalemia, mild		1,346 (23.3%)	998 (22.5%)	348 (25.8%)	0.011	764 (16.4%)	599 (16.0%)	165 (17.8%)	0.204	<0.001
Hypokalemia, severe		241 (4.2%)	162 (3.6%)	79 (5.9%)	<0.001	104 (2.2%)	75 (2.0%)	29 (3.2%)	0.040	<0.001
Hyperkalemia, mild		867 (15.0%)	541 (12.2%)	326 (24.2%)	<0.001	906 (19.4%)	586 (15.7%)	320 (34.4%)	<0.001	<0.001
Hyperkalemia, severe		207 (3.6%)	72 (1.7%)	135 (10.0%)	<0.001	168 (3.6%)	68 (1.8%)	100 (10.7%)	<0.001	0.945

^a^LOS, Length of stay; RRT, Renal replacement therapy. Data are expressed as number (%) or median (interquartile range) unless otherwise specified. Statistical analysis was performed by using a χ^2^ test, unless marked by an asterisk, in which case a Mann–Whitney *U* test was used. ^b^Before and after glucose and potassium regulation program for intensive care patients (GRIP-II) comparison. ^c^Acute Physiology and Chronic Health Evaluation II (APACHE II) scores were known for 5,294 (50.7%) patients. ^d^Acute kidney injury (AKI) was defined according to the Acute Kidney Injury Network’s Kidney Disease: Improving Global Outcomes (KDIGO) criteria. There were no data available for six (0.06%) patients. ^e^Potassium levels during the first 24 hours were known for 10,327 (98.8%) patients. ^f^Percentage of total intensive care unit (ICU) stay.

**Table 5:** **Relationship between admission hyperkalemia and acute kidney injury before and after GRIP-II** ^**a**^

	**Before GRIP-II**				**After GRIP-II**				
	**Total (** ***N*** **= 5,714)**	**no AKI (** ***n*** **= 3,798)**	**AKI (** ***n*** **= 1,925)**	***P*** **-value**	**Total (** ***N*** **= 4,609)**	**no AKI (** ***n*** **= 3,110)**	**AKI (** ***n*** **= 1,499)**	***P*** **-value**	***P*** **-value** ^**b**^
Normokalemia	4,637 (81.2)	3,330 (87.9)	1,307 (67.9)	<0.001	3,598 (78.1)	2,691 (86.5)	907 (60.5)	<0.001	<0.001
Hyperkalemia, mild	850 (14.9)	405 (10.7)	445 (23.1)	<0.001	827 (17.9)	367 (11.8)	460 (30.7)	<0.001	0.098
Hyperkalemia, severe	227 (4.0)	54 (1.4)	173 (9.0)	<0.001	184 (4.0)	52 (1.7)	132 (8.8)	<0.001	0.179

^a^Data are expressed as number (%) unless otherwise specified. Statistical analysis was performed by using a χ^2^ test. ^b^Before and after glucose and potassium regulation program for intensive care patients (GRIP-II) comparison.

**Table 6:** **Relationship between hyperkalemia and admission hyperglycemia before and after GRIP-II** ^**a**^

	**Before GRIP-II**					**After GRIP-II**					
	**Total (** ***N*** **= 5,716)**	**<15 mmol/L (** ***n*** **= 5,392)**	**15 to 20 mmol/L (** ***n*** **= 222)**	**>20 mmol/L (** ***n*** **= 102)**	***P*** **-value**	**Total (** ***N*** **= 4,611)**	**<15 mmol/L (** ***n*** **= 4,252)**	**15 to 20 mmol/L (** ***n*** **= 269)**	**>20 mmol/L (** ***n*** **= 90)**	***P*** **-value**	***P*** **-value** ^**a**^
Normokalemia	4,639 (81.2)	4,410 (81.8)	163 (73.4)	66 (64.7)	<0.001	3,600 (78.1)	3,365 (79.1)	187 (69.5)	48 (53.3)	<0.001	<0.001
Hyperkalemia, mild	850 (14.9)	778 (14.4)	43 (19.4)	29 (28.4)	0.012	827 (17.9)	718 (17.1)	66 (24.5)	33 (36.7)	0.003	0.040
Hyperkalemia, severe	227 (4.0)	204 (3.8)	16 (7.2)	7 (6.9)	<0.001	184 (4.0)	159 (3.7)	16 (5.9)	9 (10.0)	<0.001	0.511

^a^Before and after glucose and potassium regulation program for intensive care patients (GRIP-II) comparison.

Potassium variability was significantly less in patients regulated by GRIP-II during the late phase (Table 4), despite an increase of the potassium range after GRIP-II (*P* <0.001). The time in hypo- and hyperkalemia was less in both phases for patients regulated by GRIP-II, but this improvement was particularly visible after 24 hours in survivors for the time in hypokalemia.

Late mild as well severe hypokalemia decreased in patients who were regulated by GRIP-II. Mild hyperkalemia, on the other hand, increased after implementation of GRIP-II. Severe hyperkalemia did not differ before and after implementation of GRIP-II.

## Discussion

In this first study to comprehensively address the relationship of potassium concentration with outcome in the ICU, we show a strong relationship between potassium levels and potassium variability with in-hospital mortality, which persisted after adjustment for disease severity and AKI. After implementation of a novel computer-guided potassium algorithm, improvement of hypokalemia, hyperkalemia and potassium variability was observed.

The effect of our computerized regulation protocol was particularly visible during the late phase (that is, after the first 24 hours of the ICU stay). This observation underscores the fact that GRIP-II cannot affect the potassium levels that patients have upon admission to the ICU, a time when abnormal laboratory values are particularly prevalent. Thus, GRIP-II required some time to correct abnormal levels.

Although the relationship of abnormal potassium levels and potassium variability with in-hospital mortality persisted, computerized control managed to get more patients within the normal range. For those patients who still had deranged potassium levels, the mortality rate was not higher after GRIP-II than before GRIP-II. Our present retrospective study, which covered a large period that saw important changes in critical care treatment, obviously does not allow us to draw any definite conclusions on a potential beneficial mortality effect of GRIP-II, but at the least it suggests that stricter potassium control is feasible.

In contrast to a large recent observational study on the relationship between potassium and outcome [5], we took into account both sides of potassium derangements, finding an increased mortality rate in both hypo- and hyperkalemia. Hypo- and hyperkalemia are associated with an increased risk of potentially fatal complications. Both either should be avoided in critically ill patients or should be rapidly corrected when severely deranged [1–4,7]. The precise mechanisms that relate in-hospital mortality and potassium are not known. It has been proposed that mild abnormalities could be a marker of disease, whereas severe potassium derangements could be a cause of mortality [5]. Mild hypo- and hyperkalemia are often asymptomatic. Cardiac dysfunction is frequently caused by worse abnormalities.

That the multivariate relationships of both mean potassium and potassium variability with mortality (Figure 2, Table 2) were as marked as those observed by others for glucose [11] could be explained in at least two ways. One explanation could be that potassium variability has a direct causal relationship with outcome, such as through rapidly changing conditions in the cell membrane. A second explanation could be that a higher potassium variability, or, for that matter, variability of many other parameters, may be a marker of patient instability in general. Recently, it was reported that fluctuations in sodium were also associated with outcome [12]. Until more mechanistic data are available, we believe the second, noncausal explanation is more appropriate. Regardless of whether it may be useful, the GRIP system was able to decrease potassium variability.

Integration of GRIP-II into our ICU workflow was well accepted by both nurses and physicians. Because potassium regulation was integrated into an already existent glucose control protocol, it did not add any significant nursing time or costs [10]. We consider it a good example of noncritical tasks being successfully delegated to nurses and being computerized. To our knowledge, no other ICUs have yet incorporated GRIP-II, despite its being freely available on the internet. GRIP-II currently operates independently of a Patient Data Management System (PDMS), but the algorithm can also be incorporated into a PDMS. Despite safely reducing the number of patients with hypokalemia and reducing time in hypokalemia and time in hyperkalemia, GRIP-II caused a mild increase in moderate hyperkalemia. Preventing hyperkalemia and hypokalemia through GRIP-II was achieved only by regulating potassium infusion, because other actions to change potassium levels could be prescribed only by the intensivist. Thus, in cases of (impending) hyperkalemia, GRIP-II can only discontinue the potassium infusion. We assumed that this mechanism caused a higher incidence of patients with mild hyperkalemia post-GRIP, although the time in hyperkalemia decreased (Table 4). On the basis of these results, we have recently adjusted the GRIP-II target downwards slightly, from 4.3 mmol/L (in the middle of the 3.5 to 5.0 reference range) to 4.0 mmol/L.

The precise optimal range for desired potassium levels remains unknown. This has been studied in different patient cohorts, varying between 3.5 and 4.5 mmol/L [13], 4.0 and 5.0 mmol/L [14] or even 4.5 and 5.5 mmol/L in acute myocardial infarction and HF patients [15], with no consensus reached. Currently, 3.5 to 5.0 mmol/L is accepted as a safe range for ICU patients. Whether cutoff points for potassium should be more precise and could affect outcome is still unclear.

If deemed sufficiently relevant, a large prospective trial would be required to address these unanswered questions. For example, the researchers in the Normoglycemia in Intensive Care Evaluation and Survival Using Glucose Algorithm Regulation (NICE-SUGAR) study, investigated glucose control in a multicenter trial with over 6,000 patients [16].

Our study has several important limitations. A key limitation of our study is its retrospective design, so any conclusions regarding a causal effect of GRIP-II on outcome would be inappropriate. The before-after design introduces many forms of bias, in particular because many aspects of critical care have changed over the observation period, as underscored by the differences in baseline characteristics. The greatest before-after difference was the greater number of potassium measurements in patients controlled by GRIP-II, which will have affected metrics. But irrespective of the before-after character of our study, the obvious impact of close potassium monitoring by GRIP-II on the quality of regulation itself cannot be denied. We think that a potential future randomized study will be appropriate only when two computer-guided protocols are compared, as in our GRIP-COMPASS study [17], in which we compared the effect of two different computer-guided targets on atrial fibrillation after cardiac surgery.

Our potassium metrics were derived from studies on glycemic control. We considered only potassium, sex, age, severity of illness, renal function, hyperglycemia and in-hospital mortality. We also did not have APACHE II scores for an important early part of the cohort. Likewise, we did not have the access to trustworthy data about the use of drugs that could influence potassium regulation. Therefore, we were not able to take these factors into consideration.

## Conclusions

In a study unique for its scope and size, we found a clear, U-shaped relationship between early and late potassium levels and outcome. Potassium variability had a statistically independent relationship with outcome. Whether a causal relationship of variability with outcome exists is questionable. Implementation of GRIP-II led to a decrease in potassium derangements. More stringent potassium control and decreased potassium variability could influence outcome, although such an effect can be proven only in a large prospective study.

## Key messages

- For the first time, the relationship of hypokalemia, hyperkalemia and potassium variability with outcome was studied in a large, heterogeneous group of ICU patients.
- Potassium and potassium variability were strongly and independently related to outcome, similar to what has been observed for glucose.
- With the open-source GRIP-II program, potassium control was integrated with glucose control.
- GRIP-II reduced time in hypokalemia, time in hyperkalemia and potassium variability.
- Apart from the obvious practical benefits of combined computerized potassium and glucose control, it is not clear if better potassium control improves outcome.

## Abbreviations

AKI: Acute kidney injury
APACHE II: Acute Physiology and Chronic Health Evaluation II
CI: Confidence interval
GRIP-II: Glucose and potassium regulation program for intensive care patients
HF: Heart failure
ICU: Intensive care unit
IQR: Interquartile range
KDIGO: Kidney Disease: Improving Global Outcomes
LOS: Length of stay
OR: Odds ratio
PDMS: Patient Data Management System
RRT: Renal replacement therapy
SD: Standard deviation
